# Wedelolactone, a natural coumestan with multiple pharmacological effects

**DOI:** 10.3389/fphar.2025.1670032

**Published:** 2026-01-28

**Authors:** Shanshan Han, Minghe Li, Longfei Yang, Xinming Zhuang

**Affiliations:** 1 The Affiliated Hospital to Changchun University of Chinese Medicine, Changchun, China; 2 Brain Diseases Center, The Third Affiliated Hospital of Changchun University of Chinese Medicine, Changchun, China; 3 Jilin Provincial Key Laboratory on Molecular and Chemical Genetics, The Second Hospital of Jilin University, Changchun, China; 4 Department of Spinal Surgery, The First Hospital of Jilin University, Changchun, China

**Keywords:** anti-inflammatory, antimicrobial, antitumor, protective, wedelolactone

## Abstract

Natural products, especially those from medicinal plants, have been increasingly attractive to researchers. Wedelolactone (WL) is a natural coumestan that was first isolated from Wedelia Chinensis. The past decades have seen an increase in the pharmacological reports on this compound, which show that WL possesses anti-inflammatory, antiviral, antibacterial, antitumor and anti-osteoporosis activities, as well as protective effects on organ damages. This review integrates the recent progresses available on its pharmacological effects both *in vitro* and *in vivo*, and highlights its potential uses in multiple diseases.

## 1 Introduction

**FIGURE 1:**
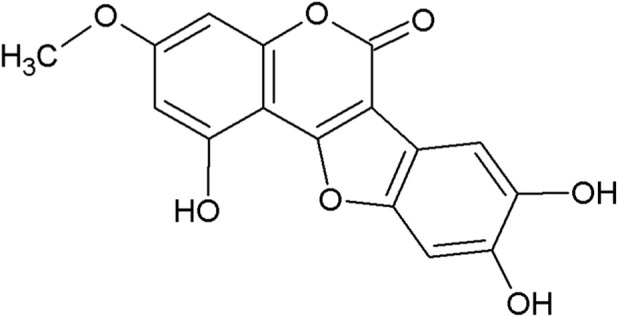
The chemical structure of wedelolactone. Molecular weight: 314.246 (CAS:524–12–9).

Since antique, natural products have been part of the medicine. The recent decades have also seen an increase in studies on natural products in the fields of infections, cancers, inflammation-related diseases (Liu et al., 2017; Roemer et al., 2011; Chen et al., 2018). A lot of natural products have been employed in clinical tests, especially in the fields of microbiological researches and cancer researches (Hassan et al., 2020). Famous examples are artemisinin and paclitaxel, to name a few. The advantages of natural products include low toxicity and multiple targets (Hassan et al., 2020). Among them, phytochemicals from plants, especially from medicinal plants, are attractive to researchers. Coumestans (benzofuran [3,2-c] coumarins) are such a kind of phytochemicals that usually exist in plants belonging to Asteraceae and Leguminosae (Ma and Zhang, 2024; Tabbasum and R, 2025). Coumestans, such as coumestrol, wedelolactone, isofraxidin, psoralidin and glycyrol, have good anti-inflammatory, anticancer, antimicrobial, antioxidant, neuroprotective and phytoestrogenic activities (Ma and Zhang, 2024; Tabbasum and R, 2025; Tu et al., 2021). For more information about coumestan compounds, please refer to these two reviews (Tabbasum and R, 2025; Tu et al., 2021). The focus of this manuscript was on the first coumestan isolated from the leaves of *Wedelia Calendulacea*, wedelolactone (C_16_H_10_O_7_, 7-methoxy-5,11,12-trihydroxycoumestan, abbreviated as WL in the later parts, the chemical structure was shown in Figure 1).

WL, as one of the major active components of *Eclipta prostrata*, has been employed as the reference compound of this herbaceous plant (*E*. *prostrata*), which is a traditional herb grown in moist and damp lands in tropical and sub-tropical climates (such as China, India and Brazil) (Saxena et al., 2015; Morel et al., 2024). Meanwhile, this is also a dietary plant in China and India (Balakrishnan et al., 2018). This herb has multiple therapeutic applications in traditional medicine, such as antidote for venomous snake bites, promoter of hair loss, and the treatment of liver cirrhosis and infective hepatitis (Mors et al., 1989; Satheesh Naik et al., 2018; Wang W. et al., 2024). In addition, WL is also produced by other species including *Wedelia Chinensis*, *Withania somnifera* and *Coldenia procumbens*. The natural sources of WL were summarized in Table 1.

**TABLE 1:** The main plants containing wedelolactone.

Plant	Genera	Parts	Content	References
*Coldenia procumbens*	Boraginaceae	Whole plants	0.18 mg/g	Rethinam and Venkatanarasimhan (2021)
*Eclipta prostrata* (*Eclipta alba*)	Asteraceae	Aerial parts	164.5 mg/100 g	Zhao et al. (2019)
*Oryza sativa*	Poaceae(Gramineae)	Seedlings	Not mentioned	Du D. X. et al. (2023)
*Wedelia Chinensis* ^a^ (*Wedelia calendulacea*)	Asteraceae	Whole plants	8.01 mg/100 g	Patil et al. (2014)
*Withania somnifera* Dunal	Solanaceae	Roots, Leaves	Not mentioned	Chauhan et al. (2022)

^a^ The first plant from which wedelolactone was isolated.

In the Chinese medicine prescription Erzhi Formula and Jiawei Erzhi Formula (Erzhi formula with *Spatholobi Caulis*, or Erzhi formula with Spatholobi Caulis and *Achyranthes bidentata* Blume) (Zhu et al., 2022), as well as in-house preparation (such as Congelex Laxative Granules prepared by Hebei Provincial Hospital of Traditional Chinese Medicine) (Qian et al., 2025), WL is also a major active component. WL is also an active component of Pet-Sang-Kard Mixed Herbal Remedy in Thailand medicine (Samee et al., 2025). Decades of researches reveals that WL possesses anti-inflammatory, antibacterial, antiviral, antioxidant, antitumor and organ-protective activities. This article reviews the latest progresses on its pharmacological effects, as well as the underlying mechanisms.

The literature research was performed by the authors until September 2025. The following databases were screened: PubMed, ScienceDirect and Web of Science. No restrictions were placed on the dates and languages of the published references to retrieve as many items regarding wedelolactone as possible. Only “wedelolactone” was used in the searching process. As for some reference that reported the isolation of WL from the same parts of the same plants, the representative one was selected and others were not included in this review. In addition, another thirty-eight reports were not included in this review because the authors used WL only as an inhibitor of IκB kinase (IKK) or casase-11 in their researches. The literature about wedelolactone was divided into groups according to their activities, such as anti-inflammatory, antibacterial, antiviral, antioxidant, antitumor and organ-protective activities.

## 2 Anti-inflammatory activities

The NF-κB pathway is critical in inflammation. In response to various inflammatory stimuli, such as pathogenic infections, and cytokines, the inhibitory protein IκBα is phosphorylated by IκB kinase α (IKKα) and IKKβ, resulting in its ubiquitination and degradation. The loss of IκBα leads to the release and nuclear translocation of NF-κB, which stimulates the transcription of inflammatory cytokines as well as other proteins (Guo et al., 2024). Since the discovery of its inhibitory activity against IKK (IC_50_: 10–20 μM), which was demonstrated by Kobori *et al* in 2004, WL thereafter was used as an anti-inflammatory agent by many groups (Kobori et al., 2004; Prakash et al., 2022). Kobori *et al* also confirmed that the inhibition of WL on LPS-induced caspase-11 expression in cultured cells (IC_50_: 35 μM) was also due to the suppression of NF-κB by WL through IKK inhibition. The inhibition on IKK of WL was even stronger than that of prostaglandin A_1_ (PGA_1_). Furthermore, due to the importance of caspase-11 in non-canonical pyroptosis, the maturation and release of the pro-inflammatory IL-1β from LPS-stimulated splenocytes could be inhibited by WL (Kobori et al., 2004). Therefore, WL has also been employed as a specific caspase-11 inhibitor in many subsequent researches (Cai R. et al., 2024; Wang Y. et al., 2020).

The work of Kobori *et al* opened the door to researches on the anti-inflammatory effects of WL. Later, WL was shown to be a G protein-coupled receptor 35 (GPR35) agonist, which may indicate its effects in allergic diseases including asthma (Deng and Fang, 2012). In a recent research, although WL has shown efficacy in asthmatic mice induced by curdlan, the effects of GPR35 agonism in this treatment have not been validated yet (Cai R. et al., 2024). In this asthmatic murine model, the neutrophil airway inflammation exacerbated by Dectin-1 agonist could be attenuated by WL, and this beneficial effect was associated with its inhibition on caspase-11, as well as its downstream chemokines such as CXCL1, CXCL3, CXCL5 and the receptor CXCR2 (Cai R. et al., 2024). These results were consistent with another report which showed that WL-containing *E*. *prostrata* extract could decrease the inflammation in a chronic allergic asthma mouse model caused by ovalbumin (Morel et al., 2024).

**FIGURE 2:**
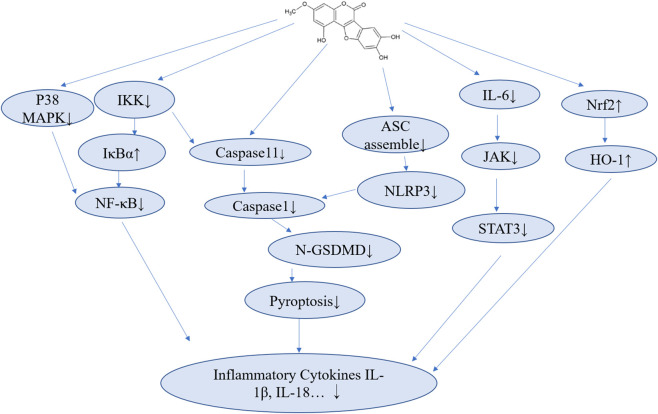
The main pathways of the anti-inflammatory effects of WL.

The anti-inflammatory effects of WL were more due to its inhibition on IKK and caspase-11. For example, in MPC-5 cells, ocular surface epithelial HCET cells (Hou et al., 2017) and murine RAW264.7 macrophages (Yuan et al., 2013), WL inhibited the inflammation via NF-κB pathway through IKK inhibition (Zhu et al., 2019). In BMDM primed with LPS, WL can block the NLRP3 inflammasome activation (through interfering ASC assembly and promoting NLRP3 phosphorylation via potentiating PKA signaling) and subsequent pyroptosis that may release pro-inflammatory IL-1β (Pan et al., 2020). The inhibition of NLRP3 activation could also be seen in leukemic marrow cells exposed to N-N′ ethylnitrosourea (N-ethyl-N-nitrosourea, ENU) (Bhattacharyya and Law, 2021). The anti-inflammatory mechanisms of WL were summarized briefly in Figure 2.

The anti-inflammatory effects of WL were also confirmed in multiple animal models. WL can attenuate the UVB (290–320 nm)-induced skin inflammation through reducing the nuclear translocation of NF-kB p65 (Ali et al., 2016). In DSS-induced colitis (a chronic inflammatory disorder of colon), the therapeutic potential of WL was associated with the inhibition of AMPK and NLRP3 (Wei et al., 2017) and downregulation of IL-6/STAT3 pathway (Prakash and Janadri, 2023) which was also suppressed by WL in indomethacin-induced colitis (Prakash et al., 2022). In fungal keratitis caused by *Aspergillus fumigatus*, WL mitigates the ocular inflammation through inhibiting neutrophil infiltration and caspase-1-mediated IL-1β maturation (Cheng et al., 2019). While in rat bacterial keratitis caused by *Pseudomonas aeruginosa*, the corneal inflammation could also be alleviated by WL via caspase-4/5/11 inhibition, which causes reduction of GSDMD-mediated non-canonical pyroptosis and reduction of subsequent active IL-1β and IL-18 (Xu et al., 2021). The anti-inflammatory effects of WL could also be seen in MSU-induced peritonitis and arthritis (Pan et al., 2020), caerulein-induced acute pancreatitis (Fan et al., 2021), collagen-induced arthritis (Cao et al., 2022), and acute kidney injury associated with severe pancreatitis (Shao et al., 2022). In a word, WL has great developing potential as an anti-inflammatory drug candidate. The anti-inflammatory mechanisms of WL both *in vitro* and *in vivo* were summarized in Table 2.

**TABLE 2:** The anti-inflammatory mechanisms of wedelolactone.

Experimental model	Inflammatory parameters assessed	Biological effects Mechanisms/actions	References
*In vitro* models			
LPS and ATP-stimulated PC12 cells	IL-1β, IL-18	Caspase-11 inhibition, GSMD cleavage ↓	Wang Y. et al. (2020)
LPS-activated microglia	IL-1β	Activation of NF-κB↓, caspase-1↓, NLRP3↓	Saker et al. (2024)
Poly (dA:dT)-induced photoreceptor 661w cells	IL-18	*Casp11* mRNA↓, *Aim2* mRNA↓	Harkin et al. (2022)
LPS-stimulated RAW264.7 cells	TNF-α, PGE2, NO	iNOS↓, COX-2↓, IκB-α degradation↑, NF-κB Activation↓	Yuan et al. (2013)
LPS-stimulated RAW264.7 cells	IL-6, TNF-α, iNOS, COX2, MCP-1	p65 nuclear translocation ↓, NF-κB activation↓, soluble epoxide hydrolase activity↓, Nrf2 pathway↑	Zhang et al. (2023)
Chondrocytes exposed to LPS-stimulated macrophage EVs	IL-1α	Caspase-11↓, GSDMD cleavage↓, pyroptosis ↓	Ebata et al. (2023)
Primary human corneal keratocytes stimulated by LPS	IL‐1β, IL‐18	Caspase-4 activation ↓, Caspase-5 activation ↓, N-GSDMD↓	Xu et al. (2021)
LPS-stimulated J774A.1 cells	IL‐1β	Caspase-1 activation↓, NLRP3 expression↓, GSDMD cleavage↓	Pan et al. (2020)
LPS-stimulated BMDM cells	IL‐1β	Caspase-1 activation↓, NLRP3 expression↓, ASC speck formation↓, GSDMD cleavage↓, PKA signaling↑	Pan et al. (2020)
LPS-stimulated HK-2 cells	IL-1β, IL-6, IL-8, TNF-α	PTPN2↑, p38 MAPK↓, NF-κB p65 phosphorylation ↓	Zhi et al. (2021)
PMA-differentiated THP-1 cells exposed to LPS	IL‐1β	Caspase-1 activation↓, NLRP3 expression↓	Pan et al. (2020)
Doxorubicin-induced inflammation in MPC-5 cells	IL-6, TNF-α, MCP-1, TGF-β	IκKα activation↓, IκBα activation↓, NF-κB p65 phosphorylation ↓	Zhu et al. (2019)
Zymosan-stimulated BMDM	IL-6, IL-10, TNF-α, IL-12	p38 MAPK activation↓, p47 phox phosphorylation↓, NADPH oxidase↓, ROS↓	Cuong et al. (2018)
LPS-activated human renal mesangial cells	IL-1β, TNF-α, NO	IκBα↑, IKKβ↓, DNA-binding activity of NF-κB p65↓	Shen et al. (2017)
*In vivo* models			
Septic acute lung injury induced by LPS	IL‐1β, IL‐18, TGF‐β, iNOS	Caspase-1↓, caspase-11↓, pyroptosis ↓, GSDMD-N↓	Zhou et al. (2024)
Sepsis-induced liver injury	IL-1β, IL-6, TNF-α	Activation of PI3K/AKT/NRF2 signaling↑, SLC7A11/GPX4↑	Yin et al. (2025)
UVB-induced skin inflammation	COX-2, NO	Activation of NF-κB↓, cGMP↓, immune cells infiltration↓, MPO↓	Ali et al. (2016)
AlCl_3_-induced neuroinflammation in rats	IL-6, TNF-α	Neuronal cell apoptosis↓	Maya et al. (2018a)
Quinolinic acid-induced induced neuroinflammation in rats	IL-1β, IL-6, TNF-α	NF-κB expression↓, neuronal cell apoptosis↓	Maya et al. (2018b)
N-methyl-N-nitrosourea (NMU)-induced inflammation in mice	IL-18	Casp1 mRNA↓, Casp11 mRNA↓	Harkin et al. (2022)
Hyperoxia-induced inflammation in mice	IL-1β, IL-18, TNF-α	Activation of Nrf2/HO-1 pathway↑	Li et al. (2024a)
Mice with systemic lupus erythematosus	IL-1β, IL-1α	Caspase 11 inhibition, GSDMD cleavage↓, pyroptosis↓, LPS leakage from gut↓	Xin et al. (2024)
Bleomycin-induced pulmonary fibrosis in mice	IL-1β, TNF-α	TGF-β1↓, AMPK activation↑, Smad2/3 phosphorylation↓	Yang et al. (2019)
Curdlan-induced asthma in mice	IL-1β, IL-1α, IL-6, IL-17A	Caspase-11 activation ↓, GSDMD cleavage↓	Cai X. et al. (2024)
Persistent inflammation in chronic wounds in mice induced by *T. nattereri* venom	IL-1α, IL-1β	Caspase-11↓, imbalance of M1/M2 macrophages↓	Lima et al. (2023)
DSS induced colitis in rats	IL-1β, IL-1α, IL-2, IL-6, TNF-α, INF-γ	IL-6/STAT3 signaling↓, Activation of NF-κB↓	Prakash and Janadri (2023)
Indomethacin-induced colitis in rats	IL-1β, IL-1α, IL-2, IL-6, TNF-α, INF-γ	IL-6/STAT3 signaling↓, Activation of NF-κB↓	Prakash et al. (2022)
Collagen-induced arthritis in mice	IL-1β, IL-6, IL-18, TNF-α	NF-κB↓, NLRP3 inflammasome activation↓	Cao et al. (2022)
N-N’ethylnitrosourea (ENU)-induced leukemia in mice	IL-1β	NLRP3 inflammasome activation↓	Bhattacharyya and Law (2021)
Acute kidney injury induced by severe acute pancreatitis in rats	IL-1β, IL-6, IL-18, TNF-α	Caspase-11 activation ↓, GSDMD cleavage↓, pyroptosis ↓	Shao et al. (2022)
Taurocholate-induced acute pancreatitis in rats	IL-1β, IL-18, TNF-α	Caspase-1↓, caspase-11↓, GSDMD-N↓, pyroptosis ↓, GPX4↑	Fan et al. (2021)
Caerulein-induced acute pancreatitis in rats	IL-1β, IL-18, TNF-α	Caspase-1↓, caspase-11↓, GSDMD-N↓, pyroptosis ↓, GPX4↑	Fan et al. (2021)
*Pseudomonas aeruginosa*-induced keratitis in rats	IL-6, IL-8, TNF-α	Caspase-11 activation ↓, GSDMD-N↓	Xu et al. (2021)
MSU-induced peritonitis in mice	IL-1β	Neutrophil infiltration↓, NLRP3 inflammasome activation↓	Pan et al. (2020)
MSU-induced arthritis in mice	IL-1β, TNF-α	Caspase-1 activation↓, leucocytes infiltration↓, NLRP3 inflammasome activation↓	Pan et al. (2020)
*Aspergillus fumigatus*-induced keratitis in mice	IL-1β	Caspase-1 activation↓, neutrophil recruitment↓	Cheng et al. (2019)
Zymosan-induced shock in mice	IL-6, TNF-α	Congestion in spleen↓, liver damage↓	Cuong et al. (2018)
DSS-induced colitis in mice	TNF-α, IL-1β, IL-17A, IL-6, IL-18	NLRP1↓, NLRP3↓, caspase-1 activation ↓	Lin et al. (2018)
DSS-induced colitis in mice	IL-1β, IL-6, TNF-α, IL-10	Caspase-1 activation ↓, NLRP 3↓, COX 2↓, iNOS↓, IκBα↑, Activation of NF-κB↓, MAPK signaling (p38, ERK, JNK) ↓, AMPK activation↑	Wei et al. (2017)
Renal inflammation in a UUO mice	IL-1β	Caspase-1 activation ↓, Caspase-11 activation ↓	Miao et al. (2018)
Hyperoxia-induced lung injury in mice	IL-1β, IL-6, TNF-α	GPX4↑	Liu et al. (2023)
Liver injury induced by sepsis in mice	IL-1β, IL-6, TNF-α	PI3K/AKT/NRF2 signaling ↑, SLC7A11/GPX4 signaling↑	Yin et al. (2025)
Hepatic inflammation induced by bile acid accumulation in mice	IL-6, TNF-α	IκBα↑, Activation of NF-κB↓, FXR activation	Wang M.-Q. et al. (2024)
Acute liver injury induced by CCl_4_ in mice	IL-1β, IL-6, TNF-α	iNOS↓, COX-2↓, NF-κB activation↓	Lu et al. (2016)

The combination of WL and luteolin produced a synergistic effect in DSS-induced colitis in mice. They strongly inhibited the expression of genes associated with IL-17 signaling pathway, such as IL-6, CCL2 and CXCL5. Specifically, in colons of mice exposed to DSS, this combination attenuated the expression of NLRP3 and NLRP1, as well as the downstream caspase-1, IL-1β and IL-18 (Lin et al., 2018). In another research, in N9 microglial cells exposed to LPS, galantamine and WL can inhibit the activation of NLRP3 inflammasome, and subsequent release of IL-1β (Saker et al., 2024).

## 3 Anti-viral effects

Great concern has been put on infections caused by viruses. AIDS represents a global threaten to public health and the effective drugs are lacking. Viral integrase is essential for the integration of viral DNA into host genome, while it does not exist in healthy host, making integrase an ideal target for developing anti-HIV drugs. In 2007, WL was demonstrated to inhibit the HIV-1 integrase, with an IC_50_ of 4 μM (Tewtrakul et al., 2007). In addition, WL can also inhibit the NS5B RNA-dependent RNA polymerase (RdRp) of hepatic C virus (HCV) by disrupting the formation of NS5B-RNA binary complex, which is crucial to the replication of HCV, one of the major viral pathogens affecting millions of people worldwide (Kaushik-Basu et al., 2008; Tseng et al., 2011; Manvar et al., 2012). The inhibition was noncompetitive to rNTP substrate while it was mainly competitive to nucleic acid template (Kaushik-Basu et al., 2008). This research also revealed a good structure-activity correlation. Later, WL was shown to inhibit the activity and the expression of NS5B and the HCV replication in MH14 cells which harbor stably replicating HCV subgenomic replicon (Manvar et al., 2012). In addition, wedelolactone can synergize with luteolin in suppressing the RdRp activity of HCV NS5B (Manvar et al., 2012). However, in Huh-7 cells undergoing HCV replication, treatment with WL increased HCV RNA, acting like IκB kinase (IKK) inhibitors such as thalidomide and 6-amino-4-(phenoxyphenenylethylamino) quinazoline (NF-κB activation inhibitor-1, NAI-1), as well as genetic manipulation of NF-κB, suggesting that direct inhibition of IKK was underlying the increased HCV replication (Rance et al., 2012; Kobori et al., 2004). Although NS5B also inhibits NF-κB signaling through blocking IKK, while in most cells, NF-κB activation can drive the production of cytokines, including those against viruses, the increase in HCV replication caused by WL in Huh-7 cells may be a consequence of balance between IKK inhibition and NS5B inhibition (Rance et al., 2012; Kobori et al., 2004).

As for human cytomegalovirus (HCMV), which present a major dander to immunocompromised people, the antiviral effects of WL were dual. First, WL can suppress the expression of immediate-early (IE) proteins, IE1/IE2, which was not due to the famous inhibitory activity of WL against NF-kB. In addition, WL can disrupt the EZH2-EED interaction, both of which are components of polycomb repressive complex 2 (PRC2) that support efficient HCMV DNA replication, thus decreasing the levels of PRC2 and PRC1 and impeding viral DNA synthesis. Furthermore, WL was non-toxic to human foreskin fibroblast cells, with a CC_50_ of 173.4 μM (Svrlanska et al., 2020).

Herpes simplex virus (HSV) can cause infections in oral cavity, nasal mucosa, brain, skin and genital areas. The effects of WL on HSV are also multifaceted. Pretreatment with WL before infection can lower the virus titres and the expression of ICP27 protein in infected cells. After absorption, wedelolactone can reduce the virus gB protein expression. WL can directly inactivate viral particles through disrupting the envelope and exposing the capsid of HSV. And this probably contributes to its inhibition on HSV-induced membrane fusion. During the interactions between HSV and host, WL could inhibit the TBK1/IRF3 pathway and block the inhibition of SOCS1 on STAT3, which are after the virus adsorption. In the mice models of HSV infections, WL can improve the disease symptoms, confirming its *in vivo* efficacy (Wang et al., 2023).

In Oropouche virus that causes Oropouche fever, WL shows potent competitive inhibitory effects on the endonuclease activity of Endo-Nter, with an IC_50_ of 310 nM that was much lower than its CC_50_ (about 373 μM). The viral replication at early post-inoculation stages could also be blocked by WL, in a concentration-dependent manner (Peinado et al., 2024a). Critical for viral replication of SARS-CoV-2, the 3C-like protease (3CL^pro^) is considered as an important target for developing antiviral drugs. In an *in vitro* natural products-based screening, WL showed potent inhibitory effects on SARS-CoV-2 3CL^pro^ with an IC_50_ of 1.003 μM (Chen et al., 2023). This inhibition was later confirmed by another group, which reported the potent inhibitory effects on SARS-CoV-2 3CL^pro^ and SARS-CoV 3CL^pro^, with an IC_50_ of 1.35 μM and 1.10 μM, respectively (Wang F. et al., 2024). They further showed that this inhibition was probably caused by an irreversible covalent binding. Its weak inhibition on other cysteine proteases indicates the good target selectivity of WL and its potential as an antiviral agent (Wang F. et al., 2024). In chikungunya virus, another positive-sense RNA virus, nonstructural protein P2 (nsP2) is thought as a preferred target for antiviral drug discovery, as this protein functions in viral replication, propagation and blocking host gene expression, among others. WL was found to be a strong inhibitor of nsP2 protease, with an IC_50_ of 2.3 μM (Peinado et al., 2024b). In chikungunya virus-infected Vero E6 cells, treatment with WL (20–30 μM) could decrease the viral titer yield (Peinado et al., 2024b). Results from metabolomics showed that WL changed the levels of lactate, myo-inositol, glucose, phosphocholine, betaine, proline, valine and phenylalanine in virus-infected cells (Peinado et al., 2024b).

## 4 Antibacterial activities

The first published report of the antibacterial activity of WL was in 2008, which showed its potent activity against *Staphylococcus epidermidis* (MIC = 15 μg/mL) and *Salmonella typhimurium* (MIC = 25 μg/mL) (Dalal et al., 2008). This was further supported by another research which also showed that WL had antibacterial activity on *Staphylococcus aureus* with MICs being 20 μg/mL (Dalal et al., 2010). However, its antibacterial effects on *Escherichia coli* and *Bacillus subtilis* were weak, with MIC being 1,000 and 500 μg/mL, respectively (Dalal et al., 2008; Dalal et al., 2010). Recently, WL was found to inhibit the biofilm of *E. coli*, with a MBIC (minimal biofilm inhibition concentration) of 17.5 μg/mL, although WL can`t inhibit the growth of *E. coli* at a concentration as high as 100 μg/mL (MIC >400 μg/mL) (Dalal et al., 2010; Buchmann et al., 2023). At 25 μg/mL, WL could completely inhibit the formation of *E. coli* biofilms (Buchmann et al., 2023). This result was consistent with a previous report that WL has strong biofilm inhibition on *E. coli* (strains PBIO729 and PBIO730) at 50 μg/mL (Stepanov et al., 2022). This compound also affects bacterial motility, chemotaxis and the biofilm extracellular matrix encasing *E. coli* cells (curli and cellulose). Genes involved in tricarboxylic acid cycle, glyoxylate metabolism (such as *glcB*) and dicarboxylate metabolism were upregulated while genes associated with arginine biosynthesis (such as *argC* and *argA*) were significantly repressed by WL (Buchmann et al., 2023).

Although WL showed weak antibacterial activities against *E. coli, P. aeruginosa* and *Shigella flexneri* (Dalal et al., 2008; Dalal et al., 2010; Buchmann et al., 2023), as mentioned above, its nanoparticles showed strong antibacterial activities against not only *E. coli*, but also *S*. *aureus*, *Pseudomonas aeruginosa* and *Klebsiella pneumonia* (Vinayagam et al., 2021). This would provide a new prospect for developing WL as an effective antibacterial agent.

As for the antifungal activity of WL, although the WL-containing methanol extract of *E. alba* has shown inhibitory effects on the growth of sorghum fungal pathogens (*Fusarium thapsinum*, *Alternaria alternata*, *Epicoccum sorghinum*, and *Curvularia lunata*) (Sollepura Boregowda et al., 2019), and that WL has therapeutic potential in *Aspergillus fumigatus* keratitis (Cheng et al., 2019), the direct evidence of the WL antifungal activity is still lacking. It is probably that WL has some, but weak, antifungal activities, since the chromatographic fractions containing only WL with high homogeneity showed inhibition on human dermatophyte *Trichophyton rubrum* strains with MIC of 500 μg/mL (Lenza et al., 2009). Its activities against other human fungal pathogens remain to be investigated.

## 5 Antivenom activities

Snakebites can cause edema, myonecrosis and hemorrhage, due to the phospholipase A_2_ myotoxins and hemorrhagic toxins within venoms (Melo and Ownby, 1999). The myotoxicity of venom could be attenuated by WL, as revealed by the inhibition of creatinocinase (CK) release from extensor digitorum longus (EDL) muscles induced by multiple kinds of snake venoms, both *in vitro* and *in vivo* (Mors et al., 1989; Melo and Ownby, 1999; Melo et al., 1994; Melo et al., 2010; Diogo et al., 2009), as well as by the reduced myonecrosis in mice (Melo and Ownby, 1999). Myonecrosis may result from damages caused by the phospholipase in venom, which can also be suppressed by WL as it inhibited crotoxin-induced hemolysis (Melo and Ownby, 1999; Melo et al., 1994; Diogo et al., 2009). In addition, WL can synergize with heparin in neutralizing the myotoxin (Melo and Ownby, 1999). The hemorrhage in EDL muscles induced by venom injection could also be reduced by preincubation with WL, which may be due to the inhibitory effects on the proteolytic activity of venom (Mors et al., 1989; Melo and Ownby, 1999; Melo et al., 1994). WL can also counteract the cardiotoxic activity of *Bothrops jararacussu* venom, exerting protective effects on rat heart (Melo et al., 2010).

In addition, WL can neutralize the venom from bees (*Africanized Apis* mellifera) (Nogueira-Souza et al., 2020). WL inhibits the phospholipase A2 and hyaluronidase *in vitro* (at 3–150 μM), and significantly decreases the CK release in EDL muscles (Nogueira-Souza et al., 2020).

## 6 Anti-oxidant activities

Multiple investigations have been performed to assess the antioxidant activity of WL, partially due to the important roles of oxidative stress in various diseases. 1, 1-Diphenyl-2-picrylhydrazyl (DPPH) and ferric reducing antioxidant power (FRAP) assays have been used to evaluate the antioxidant activity of WL, and IC_50_ of 24 μg/mL (Ding et al., 2017) and 45 μg/mL was revealed in these two assays, respectively (Ding et al., 2017; Nie and Yao, 2018). WL have demonstrated radical-scavenging activities against ⋅OH, ⋅O_2_^−^, 2,2′-azino-bis (3-ethylbenzothiazoline-6-sulfonic acid) and 1,1-diphenyl-2-picrylhydrazyl (DPPH⋅) radicals (Li et al., 2020). Besides, wedelolactone also has the ability to chelate Fe^2+^, which may be a minor branch of its antioxidant potential (Li et al., 2020). Its antioxidant activity may be due to the catechol moiety (Li et al., 2020). In one report, WL even has shown higher efficiency in removing the HOO’ radicals than the famous antioxidant Trolox (Du D. X. et al., 2023). In cells and tissues, the antioxidant activities were also confirmed by multiple groups which showed that WL activates the nuclear factor E2-related factor 2 (Nrf2)/heme oxygenase-1 (HO-1) pathway (Li K. et al., 2024; Lin et al., 2014; Li et al., 2020; Wang et al., 2022; Ding et al., 2015).

The results from *in vivo* assays were also encouraging. For example, in the mouse skin exposed to UVB, WL treatment could increase the antioxidative power (such as GSH, catalase and SOD) and decrease the oxidative load (such as H_2_O_2_, MDA), reducing the oxidative damages caused by UVB (Ali et al., 2016). In the liver tissues of mice stimulated with LPS and the acute liver injury in mice induced by CCl_4_, the oxidative stress, as well as ferroptosis, could also be lowered by WL treatment (Yin et al., 2025; Lu et al., 2016).

## 7 Anticancer activities

Many researches on the antitumor effects of WL were performed in cancer cells, which included neuroblastoma (SK-N-AS and SK-N-BE (2) cells) (Nehybová et al., 2017), hepatocarcinoma (SMMC-7721 and HepG2 cells) (Liu et al., 2012; Chen et al., 2013; Chen et al., 2015), breast cancer (MDA-MB-231 cells, 4T1 cells and MCF-7 cells) (Lee et al., 2012; Li et al., 2024b; Sarwar et al., 2020a), retinoblastoma (Y79 and Weri-Rb1 cells) (Jiang et al., 2022), prostate cancer (LNCaP, PC3 and DU145 cells) (Sarveswaran et al., 2012), leukemia (K562 and THP-1 cells) (Chen et al., 2015), melanoma (MV3) (Peng and Zhang, 2018), mantle cell lymphoma (Mino cells) (Romanchikova and Trapencieris, 2019), ovarian cancer (SKOV-3, A2780 and Ovcar3 cells) (Sarwar et al., 2020a; Luo et al., 2021; Sarwar et al., 2021), cervical cancer (Hela cells) (Sarwar et al., 2020b), bladder cancer (5637 and T24 cells) (Zhang et al., 2022), head and neck squamous cancer (SCC-4 and CU110-1 cells) (Liu et al., 2022), and multiple myeloma (RPMI-8226 and U266 cells) (Li W. et al., 2024). In most abovementioned reports, the antitumor mechanism of WL was apoptosis induction, which involves increased caspase-3 activation, PPAR cleavage, increased Bax expression, reduced expression of Bcl-2 and Bcl-xl (Jiang et al., 2022; Peng and Zhang, 2018; Tsai et al., 2009), p53 activation (Sukumari‐Ramesh et al., 2011), mitochondrial dysfunction (Jiang et al., 2022), and ROS overproduction (Jiang et al., 2022; Nehybová et al., 2017; Li et al., 2024a). During apoptosis, cell cycle arrest at G2/M phase can be induced by WL. In some cells S phase arrest occurred while in the others the proportion of S phase cells declines (Chen et al., 2015; Li et al., 2024a; Benes et al., 2011).

WL-caused ROS overproduction can also be seen in pyroptosis and ferroptosis. The activation of caspase-3 by WL can also cause the cleavage of GSDME in retinoblastoma, along with the cleavage of GSDMD by caspase-1, resulting in pyroptosis of retinoblastoma cells (Jiang et al., 2022). In multiple kinds of cancer, the aberrant activation of Keap1-Nrf2 pathway can be seen due to the activating mutation. This may be positively selected for cancers since the Nrf2 can protect cancer cells from oxidative stress. In late stage cancer patients, activation and upregulation of Nrf2 was related to resistance and poor prognosis. These make inhibiting Nrf2 signaling a target in anticancer strategies (Baird and Yamamoto, 2023). In multiple myeloma, the WL-containing *E. prostrata* extract can activate Keap1, which downregulates Nrf2 and HO-1 expression, causing GSH depletion, GPX4 downregulation, iron accumulation, lipid peroxidation, and finally ferroptosis (Li W. et al., 2024). The inhibition of Nrf2 in cancers (Li W. et al., 2024) and the upregulation of Nrf2 in normal cells and tissues (Yin et al., 2025; Li K. et al., 2024) may be due to the differences in WL concentration, cellular metabolism, and other pathways regulating Keap1-Nrf2 signaling, which may be a promising field of Nrf2 researches.

Polycomb repressive complex 2 (PRC2), composed of catalytic enhancer of zeste homolog 2 (EZH2, with methyltransferase activity), noncatalytic embryonic ectoderm development (EED) and suppressor of zeste 12 (SUZ12), is involved in tumorigenesis due to its role in the trimethylation of histone H3 at K27 (Chen et al., 2015). WL can bind to EED subunit to disrupt EED-EZH2 interaction that is critical to PRC2 activity, affecting the expression of downstream genes associated with cancers (Chen et al., 2015). Thus, in PRC2-associated cancer cells, such as HepG2 and THP-1 cells, WL has significant activities (Chen et al., 2015). The cytotoxicity of WL in Mino cells (mantle cell lymphoma) is also associated with its inhibition of histone H3K27 methylation through inhibiting EZH2 expression and reducing its histone N-methyltransferase activity (Romanchikova and Trapencieris, 2019).

In melanoma, WL inhibits Akt, leading to cyclin D1 downregulation, but increases AMPK, which may upregulate p21 that modulates cyclin D1 and PCNA associated with cell cycle (Peng and Zhang, 2018). In addition, Akt is also inhibited by WL in prostate cancer, where the AR and HER3 are also suppressed (Tsai et al., 2017a). In prostate cancers, the expression, nuclear accumulation and transcriptional activity of c-myc can also be inhibited by WL (Sarveswaran et al., 2016). Another group found that apoptosis in prostate cancer induced by WL was also JNK activation-dependent and associated with inhibition of protein kinase Cε and suppression of 5-lipoxygenase (that is important for prostate cancer survival) (Sarveswaran et al., 2012).

In breast cancer MDA-MB-231 cells, WL significantly inhibited the phosphorylation of I*κ*B-*α* after a 12-h treatment, resulting in suppression of NF-*κ*B, which, along with MAPK signaling, could suppress the invasion of tumors (Lee et al., 2012). The inhibition of I*κ*B-*α* phosphorylation appears to be time-dependent, as in a previous report an 8-h treatment did not cause an obvious inhibition of I*κ*B-*α* phosphorylation in MDA-MB-231 cells (Benes et al., 2011). The inhibition of NF-*κ*B by WL in breast cancer cells is also associated with invasion and metastasis inhibition (Lee et al., 2012; Li et al., 2024b). During invasion, WL suppresses the expression of MMP and downregulates MEK/ERK signaling (Lee et al., 2012). In the metastasis of breast cancer, WL reduces the phosphorylation of Smad2/3, attenuating the epithelial-mesenchymal transition stimulated by TGF-β1 (Li et al., 2024b). Another research reported that in MDA-MB-231 cells, WL can interact with dsDNA and suppress the DNA topoisomerase II*α*, resulting in DNA damages and cell cycle arrest (Sarveswaran et al., 2012; Benes et al., 2011). Later, the DNA damage and topoisomerase II*α* inhibition of WL was found to be redox state-dependent in cells (Benes et al., 2012). In addition, WL was also been identified as a copper-independent inhibitor of proteasome that degrade proteins, contributing to, at least partly, its cytotoxicity against breast cancer cells (Nehybová et al., 2017).

**FIGURE 3:**
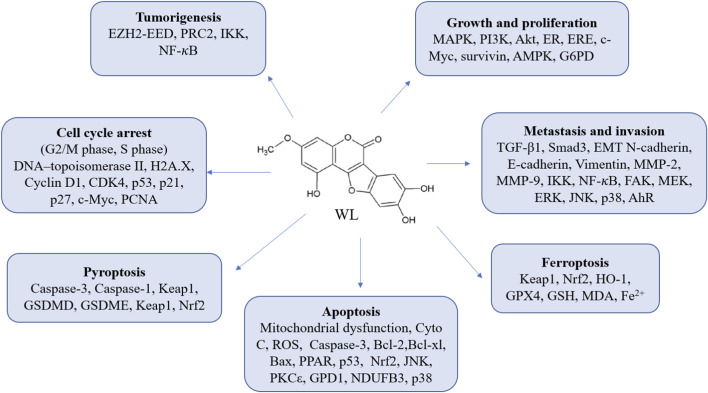
The anticancer mechanisms of WL in various cancer cells.

The anticancer effects of WL in bladder cancer may be associated with the allosteric activation of metabolic enzyme glycerol 3-phosphate dehydrogenase 1 (GPD1), as both overexpression of GPD1 and WL-mediated activation of GPD1 can suppress the bladder cancer growth *in vitro* and *in vivo* (Zhang et al., 2022). GPD1 stimulates Ca^2+^ influx and apoptosis through lysoPC-PAFR-TRPV2 pathway (Zhang et al., 2022). While in gynecological tumor cells (such as Hela cells, SKOV-3 cells, and endometrial RL95-2 cells), WL was identified as a natural inhibitor of NADH: ubiquinone oxidoreductase subunit B3 (NDUFB3), which contributed to the activation of p38 MAPK pathway and the subsequent apoptosis of these cancer cells (Li et al., 2024a). The anticancer effects of WL in various cells were summarized in Table 3. The mechanism were simply illustrated in Figure 3.

**TABLE 3:** The anticancer effects of WL.

Cancer type	Cell lines	Concentration (IC_50_)	Effects	Mechanisms	References
Cervical cancer	HeLa	5–80 μM (22.3 μM)	Apoptosis	Cell viability↓, ROS↑, G0/G1 phase cells↑, S phase cells↓, NDUF3B↓, p38 phosphorylation↑	Li et al. (2024b)
Ovarian cancer	SKOV3	5–80 μM (19.22 μM)	Apoptosis	Cell viability↓, ROS↑, G0/G1 phase cells↑, S phase cells↓, NDUF3B↓, p38 phosphorylation↑	Li et al. (2024b)
Endometrial cancer	RL95-2	5–80 μM (23.52 μM)	Apoptosis	Cell viability↓, ROS↑, G0/G1 phase cells↑, S phase cells↓, NDUF3B↓, p38 phosphorylation↑	Li et al. (2024b)
Prostate cancer	PC-3	10–30 μM	Apoptosis	Survivin↓, Cyclin D1↓, Aurora B↓, CDK4↓, ATF3↑, c-myc expression and activity ↓	Sarveswaran et al. (2016)
Prostate cancer	LNCaP	5–30 μM	Apoptosis	DNA damage↑, PARP cleavage↑, c-JNK activation↑, PKCε↓, 5-lipooxygenase inhibition	Sarveswaran et al. (2012)
Prostate cancer	LNCaP	10–30 μM	Apoptosis, invasion	DNA degradation↑, Survivin↓, Cyclin D1↓, Aurora B↓, CDK4↓, ATF3↑, c-myc expression and activity ↓	Sarveswaran et al. (2016)
Mammary carcinosarcoma	Rat W256 cells	0.01–30 μM; (26.9 μM)	Apoptosis	Cell viability↓, caspase-3 activation ↑	Idris et al. (2009)
Neuroblastoma	SK-N-AS	6.25–100 μM; (16.2 μM)	Apoptosis	caspase-independent cell death, NF-kB activation↓	Sukumari‐Ramesh et al. (2011)
Breast cancer	MDA-MB-231	10, 20, 30 μM	Apoptosis	S and G2/M phase arrest ↑, DNA damage ↑, interacts with dsDNA, DNA topoisomerase Iiα inhibition, p-Akt↑, p-p53↑	Benes et al. (2011)
Breast cancer	4T1	10–40 μM	Proliferation, invasion, migration	p-Smad2/3↓, MMP2↓, MMP9↓, Vimentin↓, E-cadherin↑	Li et al. (2024b)
Breast cancer	MDA-MB-231; T47D	25 μM	​	Lysosomal Cu^2+^↑, lysosomal membrane permeability↑, oxidative stress↑, GSH↓	Kučírková et al. (2018)
Breast cancer	MDA-MB-231	5–20 μM	Invasion and migration↓	Cell viability↓; MMP2↓, MMP9↓, I*κ*B-*α*/NF-*κ*B ↓; MEK/ERK signaling↓; FAK↓	Lee et al. (2012)
Liver cancer	HepG2	3.125, 12.5, 50 μM	Apoptosis, Invasion↓	Cell viability↓, G2/M phase arrest ↑, EZH2-EED interactions ↓, PRC2 degradation ↑	Chen et al. (2015)
Leukemia	K562	3.125, 12.5, 50 μM	Apoptosis	Cell viability↓, S phase cells ↑, EZH2-EED interactions ↓, PRC2 degradation ↑	Chen et al. (2015)
Leukemia	THP-1	3.125, 12.5, 50 μM	Apoptosis	Cell viability↓, EZH2-EED interactions ↓, PRC2 degradation ↑	Chen et al. (2015)
Melanoma	MV3	5–80 μM	Invasion, migration, apoptosis	Cell viability↓, Bcl-2↓, Bax↑, cyclin D↓, p21↑, AKT↓, AMPK↑	Peng and Zhang (2018)
Mantle Cell Lymphoma	Mino cells	0.1–7.5 μM	Proliferation↓	Cell viability↓, EZH2↓, H3K27 methylation↓	Romanchikova and Trapencieris (2019)
Retinoblastoma	Y79, Weri-Rb1 cells	10–50 μM	Apoptosis, pyroptosis	Viability↓, ROS↑, PARP cleavage ↑, caspase-3 activation↑, Bax↑, Bcl-2↓, caspase-1 activation↑, GSDMD cleavage↑, GSDME cleavage ↑, MMP↓, cytoplasm cytochrome C↑	Yin et al. (2025)

In addition, the cytotoxic activity of WL has something to do with copper, and the interactions between WL and copper may lead to the disruption of cellular homoeostasis (Kučírková et al., 2018). WL can non-competitively and reversibly inhibit glucose-6-phosphate dehydrogenase (G6PD, IC_50_: 3.64 μM), which is upregulated in cancers and the impaired activity of which can limit cell proliferation, suggesting its potential in cancer treatment (Luo et al., 2021).

The combination of WL and other anticancer agents also showed good efficacy in several kinds of tumors. WL can potentiate the effects of IFN-γ in inducing apoptosis in tumor cells (HepG2) through prolonging the STAT1 activation by inhibiting its dephosphorylation mediated by TCPTP (T-cell protein tyrosine phosphatase) that deactivates STAT1 (Chen et al., 2013). In addition, WL has also been shown to enhance the efficacy of luteolin and apigenin in androgen receptor (AR)-positive prostate cancer in xenografted mice (Tsai et al., 2009). The mixture of these three compounds function synergistically to suppress the AR activity and the *W. chinensis* extract containing them showed efficacy in castration-resistant prostate cancer xenograft model through inhibition of AR activity and HER2/3 pathway (Tsai et al., 2017a). This extract can also enhance the therapeutic efficacy of docetaxel in prostate cancer (through reducing chemokines and altering the tumor microenvironment) and can lower the toxicity of docetaxel (Tsai et al., 2017b). Through downregulation of c-myc expression and activity, WL can also synergize with enzalutamide in inducing apoptosis in prostate cancer (LNCaP cells) (Sarveswaran et al., 2016). In cervical cancer Hela cells, WL can synergize with cisplatin in inhibiting cervical cancer cell growth (Sarwar et al., 2020b). However, in another research by this group, WL and cisplatin produced an antagonistic interaction in MCF-7 and SKOV-3 cells (Sarwar et al., 2020a). This indicates that this combination may produce different outcomes in different tissues, which need to be further explored. A later report of this group also found that the sequential addition of WL and cisplatin can produce additive effects in ovarian cancer cells (Sarwar et al., 2021). This combination could increase the intracellular accumulation of cisplatin while the *in silico* tests suggested WL inhibits many epigenetic factors associated with tumor development (Sarwar et al., 2021).

Due to its low bioavailability (although the bioavailability of WL could be improved by other compounds in *Wedelia chinensis* extract (Tsai et al., 2015), they may increase other risks) (Tsai et al., 2009), effects have been made to improve its bioavailability, such as encapsulating indocyanine green (ICG) and WL into liposomes, which is photoreactive and activated by near-infrared light (Zhang et al., 2016). This strategy largely improves the solubility, bioavailability and anti-tumor efficacy (against HepG2 xenografts) of WL in mice (Zhang et al., 2016). Later, this group also reported a synergistic drug delivery system combining chemotherapy and photothermal therapy through coating wedelolactone liposome with gold nanoshell (AuNS-Wed-Lip), attenuating the shortcomings of traditional liposomes (Zhang et al., 2019a). This group further developed another graphene oxide-based trimodal synergistic drug delivering system (chemotherapeutic/photothermal/photodynamic, composed of ICG-WL-graphene oxide) controlled by near-infrared light (Zhang et al., 2019b). This trimodal synergistic therapy showed excellent efficacy in both Hela cells and tumor-bearing mouse models (Zhang et al., 2019b). In addition, the uptake, retention and intracellular release of WL in cancer cells could also be improved by encapsulating WL with PLGA nanoparticles (Das et al., 2019). Through SOX2 and ABCG2 inhibition, this encapsulation by nanoparticles can sensitize tumor cells to paclitaxel in chemoresistant breast tumor stem cells. This strategy also gave encouraging results *in vivo* (Das et al., 2019).

The anti-tumor efficacy of WL in animals has been shown in multiple researches (Li et al., 2024b; Peng and Zhang, 2018). In xenograft nude mice with melanoma cells (Peng and Zhang, 2018), prostate cancer cells (Sarveswaran et al., 2016), breast cancer cells (Li et al., 2024b; Das et al., 2019), bladder cancer cells (Zhang et al., 2022), retinoblastoma cells (Jiang et al., 2022), multiple myeloma cells (Li W. et al., 2024), the tumor growth could be suppressed by WL treatment. The *in vivo* results also confirmed the synergy between WL and enzalutamide in suppressing bladder cancers (Sarveswaran et al., 2016).

## 8 Renal-protection

The protective effects of WL on kidney were reported in several aspects, such as in LPS-induced injuries, in renal fibrosis and in xenobiotics-induced damages. In HK-2 cells, WL can protect the renal cells from LPS-caused injuries by inhibiting inflammation and blocking apoptosis through increasing the expression of protein tyrosine phosphatase non-receptor type 2 (PTPN2) that may regulates P38 MAPK/NF-κB pathway (Zhi et al., 2021). While the enhanced proliferation of human renal mesangial cells (HRMC) caused by LPS exposure and the production and release of inflammatory factors, such as IL-1β, TNF-α and NO, from HRMC, could also be suppressed by WL through IKK and NF-κB inhibition (Shen et al., 2017). In rat renal tubular epithelial NRK-52E cells, angiotensin-induced expression of renal fibrosis markers (such as TGF-β, fibronectin and collagen I), can be suppressed by WL via caspase-11 inhibition (Miao et al., 2018). In mice subjected to unilateral ureteral obstruction, WL can also inhibit the expression of IL-1β, TGF-β, fibronectin and collagen I in kidney through inhibiting caspase-11, improving the histological changes associated with renal fibrosis (Miao et al., 2018).

In the doxorubicin-caused kidney injury which involves oxidative stress and inflammation, WL exerts its nephroprotective effects through two manners: antioxidative activity and anti-inflammatory activity. On one side, WL can increase the SOD, CAT and GSH-Px activity and suppress the ROS and MDA level in mouse podocyte clone 5 (MPC-5) cells; on the other side, WL can lower the levels of inflammatory cytokines through IκK/IκB/NF-κB signaling (Zhu et al., 2019). However, the poor solubility and low bioavailability limit its use. The newly-developed WL-contained micelles composed of Solutol^®^ HS15 and lecithin, can improve the permeability and bioavailability significantly while keep its ability to mitigate the renal injury caused by doxorubicin (Feng et al., 2019). In mice model of lupus nephritis, WL could effectively mitigate the renal damage through suppressing caspase-4/5/11-mediated noncanonical pyroptosis in macrophages (Liu et al., 2025).

WL, as a competitive inhibitor of organic cation transporter 2 (OCT2) that mediates the uptake of cisplatin, has been reported to alleviate the damages of OCT2-overexpressing HEK293 cells caused by cisplatin and to reduce the cisplatin-induced renal damages in ICR mice through lowering its accumulation in kidney (Wang et al., 2021). The aristolochic acid (AA) in Aristolochia, and Asarum genera can cause damages to kidney through the uptake of AA by proximal tubule cells mediated by organic anion transporter 1 (OAT1) and OAT3. WL can strongly inhibit both OAT1 and OAT3 in the OAT-mediated 6-CF uptake assays, with both IC_50_ less than 10 μM (Li C. et al., 2020). In mice treated with AA, the renal damages could be significantly lowered by WL, as evidenced by reduced levels of serum creatinine and blood urea nitrogen, as well as lowered necrosis and tubular dilation in kidney (Li C. et al., 2020). The inhibition of OAT1 and OAT3 by WL may also increase the system exposure of other drugs such as furosemide and cephardine since the OAT inhibitor probenecid can increase it (Tweedie et al., 2013). In addition, OCT2 inhibition by WL may decrease the system exposure of dofetilide, as it is decreased by OCT2 inhibitor cimetidine (Tweedie et al., 2013). Therefore, when these drugs and WL-containing herbs were given together, these interactions should be considered.

## 9 Glucose and lipid metabolism

Obesity, which is triggered by adipose tissue accumulation that originates from adipogenic differentiation of precursor cells and adipocyte hypertrophy, is a risky factor for many diseases including diabetes mellitus. In human adipose tissue-derived mesenchymal stem cells exposed to adipogenesis-inducing medium, WL can suppress the lipid droplet formation and adipogenic differentiation through sustaining ERK activation, which causes PPAR-γ inhibition (that is essential for adipogenesis) and lowers the expression of CEBP-α, lipoprotein lipase and adipocyte fatty acid-binding protein (aP2) (Lim et al., 2012). Its anti-obesity activity is estrogen and androgen independent (Lim et al., 2012). Later in DIO mice, WL, in safe dosages that are wide, was found to decrease fat mass, body weight gain and to increase the adipose browning and energy expenditure, without affecting food intake or movements. The mechanisms involve suppression of nicotinamide N-methyltransferase and subsequent activation of SIRT1/AMPK/PPARα pathway (Yao et al., 2022).

WL also showed anti-diabetic and blood glucose-regulating activities in streptozotocin (STZ)-induced Wistar albino rats. WL inhibits the α-glucosidase and α-amylase that catalyze the breakdown of the carbohydrates into monosaccharides for supplying the host for energy and metabolism. In the other side, WL can increase the lowered blood insulin level caused by STZ, further decreasing the postprandial blood glucose in rats (Kumar et al., 2018; Shahab et al., 2018). The mechanism may involve the decreased inflammatory responses and oxidative stress by WL (Kumar et al., 2018; Shahab et al., 2018).

The beneficial effects of WL in diabetes are also shown in islet protection. The insulin-producing islet β cells were protected by WL from cytokine IL-1β-induced inflammatory damage and dysfunction in zebrafish (Delgadillo-Silva et al., 2019). Thus, the hyperglycemia in zebrafish was ameliorated by WL. In cultured human and mouse islets, the protection of WL against cytokines-induced apoptosis was also confirmed (Delgadillo-Silva et al., 2019). The protection of WL in rats against STZ-caused damages in β cells may also be through reducing the toxic advanced glycation products (Shahab et al., 2018). Later, the gold nanoparticles of WL were synthesized, which can protect rat islet-derived RIN-5F cells from di-(2-ethylhexyl) phthalate (DEHP)-caused damages and dysfunction of insulin secretion through scavenging free radicals, upregulating anti-apoptotic proteins and improving insulin secretion (Ramachandran et al., 2019). The anti-diabetic effects of WL gold nanoparticles were also confirmed in DEHP-treated rats, as revealed by the lowered blood glucose, increased insulin and liver glycogen content (Ramachandran et al., 2019). WL-containing *C*. *procumbens* methanol extract also showed anti-diabetic activity in rats treated with STZ (Rethinam and Venkatanarasimhan, 2021).

The changes of lipid profile in diabetic rats induced by STZ, such as elevated levels of triglycerides (TG), total cholesterol (TC), LDL, VLDL and lowered level of HDL, can be reversed by WL (Kumar et al., 2018). This was consistent with another research, which reported that in high-fat diet (HFD)-induced hamsters, WL can inhibit the rise in blood levels of TG, TC and LDL-cholesterol (Zhang et al., 2015). The mechanism study revealed that the increased activation of AMPK and PPARα, may be responsible for these effects. In mice exposed to Triton WR-1339, WL also decreases the TG levels (Zhang et al., 2015).

## 10 Bone-related diseases

The reported effects of WL on bones are mainly on osteoporosis and osteoarthritis (OA). Early in 2006, the anti-osteoporotic effects of *W*. *calendulacea* Less. has been documented, as evidenced by the improved biomechanical and biochemical parameters, and WL was speculated as responsible for this activity (Annie et al., 2006). At concentrations causing no significant cytotoxicity to RAW264.7 cells, WL can inhibit the osteoclastic differentiation induced by receptor activator of nuclear factor κB ligand (RANKL) through NF-κB/c-fos/NFATc1 signaling (Liu et al., 2014; Liu et al., 2016a). It can also suppress breast cancer (MDA-MB-231)-mediated osteoclastogenesis, which involves the suppression of Akt/mTOR pathway (Hsieh et al., 2015). In addition, by downregulating macrophage colony stimulating factor (M-CSF) in osteoblasts stimulated by MDA-MB-231 cells, WL can regulate the interaction of osteoblasts and osteoclasts, adding beneficial points to the effects of WL on osteoporosis mediated by breast cancer (Hsieh et al., 2015). In osteoclastic RAW264.7 cells, the inhibition of osteoclastogenesis is associated with increased complex formation of plexinA1 with Nrp1, rather than DAP12, and reduced PLCγ2 activation (Liu et al., 2016b). Semaphorins regulate osteoblasts and osteoclasts through forming complexes with plexins to maintain the balance between resorption and formation of bone (Deng et al., 2018). The Sema4D and Sema7A production in RAW264.7 cells induced by RANKL, as well as the formation of Sema4D-PlexinB1 complex, can also be inhibited by WL, causing suppression of osteoclastogenesis (Deng et al., 2018). In mouse model of calvarial osteolysis induced by polystyrene particles, WL was shown to decrease the size and number of pores, and number of osteoclast around bone, improving bone mineral density (Lu et al., 2022).

The other side of bone homeostasis is osteoblastogenesis, which is enhance by WL in BMSC (Liu et al., 2016a; Liu et al., 2016b). In BMSC, WL can stimulate the binding of plexin A1 with Nrp1, which activates the downstream Wnt/β-catenin signaling that facilitates osteoblastogenesis (Liu et al., 2016a; Liu et al., 2016b); WL also increase the production of Sema3A, which may result in the binding of plexin A1 with Nrp1 (Liu et al., 2016a). Meanwhile in BMSC, WL can increase Sema7A, reduce Sema3E expression, and facilitate the Sema7A-PlexinC1-Beta1 complex (Deng et al., 2018). In BMSC, the positive effects of WL on osteoblastogenesis can also be exerted through increasing phosphorylation of ERK and JNK, which enhances the expression of bone morphogenetic protein (BMP2, essential biomolecules mediating osteoblastogenesis) and the phosphorylation of Smad1/5/8 (Zhu et al., 2018). Recently, WL was found to increase the expression of methyltransferase METTL3, which mediates the m6A (N6-Methyladenosine) methylation modification of mRNA of genes associated with osteoblastogenesis such as *Osterix* and *Osteocalcin*, facilitating the osteoblastogenesis (Tian et al., 2023). The most direct support for the usage of WL in osteoporosis came from ovariectomized mice, where the ovariectomy-induced bone loss can be mitigated by WL treatment through suppressing osteoclast activity and facilitating osteoblast differentiation (Deng et al., 2018).

The osteoinductive hydroxyapatite nanoparticles (∼20 nm) can enhance the positive effects of WL on osteoblastic differentiation from BMSCs, mineralization and the expression of osteoblastogenesis-related genes (such as osteorix, Runx2 and osteocalcin) (Dong et al., 2019). The addition of oleonuezhenide can also be beneficial by reversing the ERK phosphorylation caused by WL in BMSC and by increasing Wnt5a and CK2α expression. Therefore, the combination of WL and oleonuezhenide may have less cytotoxicity and better osteoblastogenesis and bone mineralization. In ovariectomized mice, the bone loss was alleviated by this combination through elevating osteoblastogenesis (Deng et al., 2019).

As for OA, WL can boost the chondrogenic differentiation of rat mesenchymal stem cells through upregulating FOXO1 by inhibiting enhancer of zeste homolog 2 (EZH2) that promotes the histone H3 lysine 27 trimethylation of FOXO1 promoter region (Qin et al., 2023). In addition, WL can also suppress the expression of the miR-1271-5p that can inhibit post-transcriptionally the expression of FOXO1 through binding to FOXO1 3′-UTR (Qin et al., 2023). Inflammation and extracellular matrix (ECM) degradation contribute to the pathogenesis of OA. In human primary chondrocytes, WL can inhibit the production of inflammatory mediators induced by IL-1β through inhibiting NF-κB pathways. In addition, the IL-1β-caused ECM degeneration, as revealed by the lowered levels of SOX9 and collagen II, as well as the increased expression of Adamts5, MMP1, MMP3 and MMP13, can also be mitigated by WL. Furthermore, WL was confirmed to ameliorate the cartilage degeneration in the mouse medial mensius model of OA (Sun et al., 2024).

## 11 Neuroprotective activities

In general, antioxidants have been considered as beneficial for brain that is vulnerable to oxidative stress due to the richness in substrates of oxidative reactions (such as unsaturated fatty acids) and less antioxidant power in brain (Lin et al., 2014). In a mouse neuronal damage model induced by D-galactose, WL can facilitate the translocation and activation of Nrf2, increasing the expression of γ-glutamyl-cysteine synthetase (γ-GCS). These may attenuate the increased MDA and apoptosis and loss of neuronal cells in cerebral cortex of mice caused by D-galactose (Lin et al., 2014). The antioxidative activity of WL also contribute to the neuroprotection against aluminium-induced neurodegeneration in rats (Maya et al., 2018a) and neurotoxicity induced by quinolinic acid in rats (Maya et al., 2018b). In the rat model of sporadic amyotrophic lateral sclerosis, the neurodegenerative damages of motor neurons induced by aluminium can be alleviated by WL through multiple facets: improvement of antioxidant status, increase in BDNF level, attenuation of glutamate excitotoxicity, inhibition of neuronal apoptosis, and suppression of inflammation (Maya et al., 2018a). Many of these effects were also seen in rats exposed to quinolinic acid, whose toxicity can impair motor function and motor learning capacity (Maya et al., 2018b). In this quinolinic acid-induced model, WL has also shown the protection against neuronal damages in histopathological assessments (Maya et al., 2018b).

The accumulation of α-synuclein in substantia nigra region, degenerative changes of dopamine neurons, and abnormal lipid metabolism are significant changes in the neurodegenerative Parkinson disease (PD) (Sharma et al., 2021). WL treatment can reduce α-synuclein level, improve neuronal health and behavior, dopamine level, and lipid metabolism in a worm model of PD. The neuroprotection was associated with alleviation of oxidative stress and improvement of mitochondria through NRF2/SKN1 pathway (Sharma et al., 2021).

The neurodegenerative damages of photoreceptor cells due to oxidative stress and inflammation caused by NMU, can be alleviated by WL through the inhibition of Aim2 inflammasome associated pathway (Harkin et al., 2022). In addition, WL can also be protective in DNA-induced death in photoreceptor cells (Harkin et al., 2022; Harkin et al., 2018).

Tropomyosin receptor kinase B (TrkB, viz. NTRK2 (abbreviation for neurotrophic tyrosine kinase receptor 2)), can be upregulated pathologically in central and peripheral nervous system during neurological diseases such as Alzheimer’s disease (AD) and PD, making it a potential target for treating these diseases (Hakami et al., 2024). *In silico* tests showed that WL may inhibit TrkB through docking in its ATP-binding pocket (Hakami et al., 2024), suggesting its potential use in AD and PD. WL may inhibit the druggable enzymes (acetylcholinesterase (AChE) and butyrylcholinesterase (BChE), which are associated with formation of amyloid plaques) in AD, according to results from molecular docking assay (Du Y.-W. et al., 2023). Its neuroprotection may also involve antiradical and Cu(II)-chelating activities (Du D. X. et al., 2023).

The WL-containing methanol extract from *E. alba* could alleviate the epilepsy-like symptoms induced by picrotoxin through modulating GABA_A_ receptors rather than GABA release from synapses (Sathaye et al., 2013). This was consistent with the reported binding activity of WL to central benzodiazepine site of GABA_A_ receptor complex (Pocas et al., 2006).

## 12 Cardiovascular protection

WL can suppress the CK release from rat hearts administrated with *B. jararacussu* venom, and inhibit the decrease of cardiac tension and other functional parameters of heart, in a Langendorff perfusion model (Melo et al., 2010). In primary cultured rats myocardial cells, pre-administration with WL can facilitate the survival of cells experiencing anoxia/reoxygenation, possibly through increasing Bcl-2 expression and decreasing Bax and PARP expression (Xu et al., 2012). The coronary risk factor and atherogenic index augmented in STZ-treated rats can be reduced by WL, suggesting a protection for cardiovascular system (Kumar et al., 2018).

Anomalous expression of endothelin-1 subtype B (ET_B_) receptor in vascular smooth muscle cells (VSMC) can be associated with vasoconstriction and proliferation in multiple cardiovascular diseases (Dimitrijevic et al., 2009). High glucose or diabetes can upregulate VSMC ET_B_ receptor through ERK1/2 or P38 MAPK-NF-κB signaling pathway. As a NF-κB inhibitor, WL can suppress the expression of ET_B_ receptor and ET_B_-mediated vasoconstriction in *ex vivo* assays with rat superior mesenteric arteries (Dimitrijevic et al., 2009). Additionally, WL can also suppress vascular remodeling and neointimal hyperplasia in rats with balloon injury in left common carotid artery. In this model, WL inhibits Akt signaling and upregulates AMPK pathway, which may further induce p21 expression and inhibit cyclin D1 expression, resulting in cell cycle arrest and growth inhibition of VSMC (Peng et al., 2017).

## 13 Hepato-protective activities

Hepatic diseases, including drug-induce liver damages, fibrosis and cholestasis, seriously influence public health (Luo et al., 2018), and WL has shown multiple kinds of protection for liver. In primary hepatocytes isolated from rats, WL showed protection against phalloidin (one of the strongest liver poisons), D-galactosamine or CCl_4_-induced hepatotoxicity, at a concentration of 10 μg/mL (Wagner et al., 1986). The increase in lactate dehydrogenase (LDH), glutamic oxaloacetic transaminase (GOT) and glutamate pyruvic transaminase (GPT), which are markers of liver damages, induced by CCl_4_ could be decreased by wedelolactone (Upadhyay et al., 2012). Moreover, the inhibition of hepatic microsomal drug metabolizing enzymes and the loss of hepatic lysosomal acid and alkaline phosphatase induced by CCl_4_ could be counteracted by WL (Upadhyay et al., 2012). In HepG2 cells exposed to CCl_4_, the viability could be elevated by WL treatment (Giang et al., 2024). This kind of hepatoprotection was further confirmed in CCl_4_-induced damages in rats and mice, which employed more biochemical parameters (such as hexobarbitone-induced sleep time, zoxazolamine sleep time, BSP clearance, blood GPT and bilirubin) to demonstrate the protection (Singh et al., 2001; Lu et al., 2016). The mechanisms involve the inhibition of hepatic inflammation and apoptosis of liver cell, and the increase in antioxidative capacity (Lu et al., 2016). The protection against D-galactosamine-induced liver injury was also confirmed in mice, through lowering the oxidative stress and inflammatory responses (Wang W. J. et al., 2020) In the paracetamol-induced rat model of liver damage, the elevated levels of GPT, GOT, alkaline phospholipase, and total bilirubin in blood could be lowered by WL, while the anatomical architecture could be recovered, and the bioavailability of paracetamol was not affected (Sagar et al., 2006). This may be useful in conditions that require prolonged paracetamol therapy and lowered paracetamol toxicity. In zebrafish exposed to thioacetamide that dysregulates hepatic lipid metabolism, the liver injury and liver fat accumulation can be ameliorated by WL through improving steroid biosynthesis and fatty acid metabolism, as revealed by spatial metabolomics and transcriptomics analysis (Chen et al., 2024). In sepsis-induced liver injury caused by caecal ligation and puncture in mice, the hepatic damages from oxidative stress and inflammatory responses, could be alleviated by WL through activating PI3K/AKT/Nrf2 and SLC7A11/GPX4 pathways (Yin et al., 2025).

In the mouse model of concanavalin A-induced hepatitis (immune-mediated liver injury), pretreatment with WL also demonstrated hepatoprotection, as shown by the decreased concentrations of blood transaminases and liver injury (Luo et al., 2018). The mechanisms involve the reduced levels of TNF-α, IFN-γ, IL-6 in blood and the lowered gene expression of *CXCL10* and intercellular adhesion molecule 1 (*ICAM1*) in liver, and the attenuated leukocytes infiltration and T-cell activation, which may result from the inhibition of NF-κB signaling pathway (Kobori et al., 2004; Luo et al., 2018). In a mouse model of liver injury induced by α-naphthylisothiocyanate (ANIT), WL exerts its anti-cholestatic/hepato-protective effects through reducing hepatic bile acid accumulation and regulating bile acid transportation and metabolism via FXR activation. The secondary oxidative stress and inflammatory responses in liver caused by excessive intrahepatic bile acid accumulation can also be mitigated by WL through NF-κB inhibition and NRF2 activation (Wang M.-Q. et al., 2024).

Anti-fibrotic effects of WL were mediated by inducing apoptosis through increasing Bax and decreasing Bcl-2, in LX-2 cells which is an activated human hepatic stellate cell line (Xia et al., 2013). The increased activation of ERK and JNK and the decrease of NF-κB activation induced by TNF-α also contribute to the anti-fibrotic activity of WL (Xia et al., 2013). In these LX-2 cells stimulated with TGF-β, the activation of Hippo/YAP/TAZ pathway that triggers liver fibrosis, can also be inhibited by WL (Zhang et al., 2024). In mice hepatic fibrosis induced by bile duct ligation (BDL), WL is also protective through inhibiting HSC activation mediated by TGF-β/Smad signaling (Ai et al., 2021). Another research found that YAP suppression was also involved (Zhang et al., 2024). They also found that in mice treated by CCl_4_, the liver fibrosis can be inhibited through suppressing Hippo/YAP/TAZ signaling (Zhang et al., 2024). Its low intestinal absorption could be increased by forming phyto-vesicles with phosphatidyl choline, which also increases the hepatoprotective activity in CCl_4_-treated hepatocytes (Kobori et al., 2004). Additionally, WL and schisandrol B can produce synergistic effects on reversing the hepatic fibrosis in mice induced by CCl_4_ (Ai et al., 2021).

In addition, WL has demonstrated antiviral activities against HCV, a human pathogen often closely associated with steatosis, cirrhosis and hepatocellular carcinoma, through disrupting the formation of NS5B-RNA complex where NS5B is required for viral RNA replication (Kaushik-Basu et al., 2008; Manvar et al., 2012), providing another kind of hepatoprotection. This may be the material base underlying the traditional use of *E. alba* for infective hepatitis (Wagner et al., 1986). Its inhibition of hepatocellular carcinoma may also add to the hepatoprotection of WL (Pan et al., 2021). In rats fed with high-fatty diet, the inhibitory effects on the microvesicular fat deposition, mononuclear infiltration, necrosis of liver, as well as stimulatory activity on hepatocyte regeneration, have also been demonstrated by WL-containing extract, which was consistent with the traditional therapeutic efficacy of *E. alba* on liver cirrhosis (Satheesh Naik et al., 2018; Wagner et al., 1986).

## 14 Lung protection

WL can save the viability of lung cells *in vitro* challenged with hyperoxia (Li K. et al., 2024), and cigarette smoking extract (CSE) (Ding et al., 2015; Ding et al., 2018). In normal human bronchial epithelial (NHBE) cells, the oxidative damages and inflammation induced by CSE can be mitigated by WL through activating Nrf2 pathway and inhibiting NF-κB (Ding et al., 2015). This group also found later that *E. prostrata* extract containing WL and demethylwedelolactone as the major components, can block the excessive autophagy flux activation that is important in the CSE-induced NHBE injury (Ding et al., 2018).

Hyperoxia-induced acute lung injury (ALI) in mice, which involves oxidative stress, inflammation and ferroptosis, can be alleviated by WL through activating Nrf2/HO-1 signaling and blocking ferroptosis in lung. The protection also involves inhibition of apoptosis and reduce in the inflammatory cytokines in lung tissues induced by hyperoxia (Li K. et al., 2024; Liu et al., 2023). In septic ALI in mice induced by LPS, WL treatment can improve the pulmonary injury, edema and inflammation through reducing pyroptosis and M1 polarization of alveolar macrophages (Zhou et al., 2024). While another group showed that in LPS-induced ALI in mice, WL bound and suppressed the overexpressed soluble epoxide hydrolase (leading to increased levels of epoxyeicosatrienoic acids that own anti-inflammatory and antioxidative activities) and inhibited macrophage activation. The WL-soluble epoxide hydrolase interactions can further inhibit GSK3β by increasing phosphorylation at Ser 9, resulting in NF-κB inactivation and Nrf2/HO-1 activation that relieve inflammation and oxidative stress (Zhang et al., 2023).

In rat pulmonary arterial hypertension (PAH) induced by monocrotaline, WL can alleviate the symptoms associated with PAH (such as right ventricular hypertrophy and pulmonary arterial remodeling), through inhibiting pyroptosis mediated by caspase-11 (Wu et al., 2022). WL can exert its antifibrotic activity in mouse pulmonary fibrosis induced by bleomycin through increasing AMPK activation and reducing TGF-β1 overexpression and Smad2/3 phosphorylation, and downstream Raf1/MAPK signaling (Yang et al., 2019). To improve the delivery efficacy of WL, this group further developed WED-load immunoliposomes modified with pulmonary surfactant A monoclonal antibody, which showed promising potential in treating pulmonary fibrosis (Zhao et al., 2023). Additionally, WL can also mitigate acute pancreatitis-associated lung injury in rats through suppression of pyroptosis and ferroptosis by upregulating GPX4 (Fan et al., 2021).

## 15 Enzyme inhibition

In 2004, WL was identified as a specific inhibitor of IκB kinase (IKK) with irreversible suppression at the level of 20–30 μM (Kobori et al., 2004). Later, WL was found to be inhibitors of multiple enzymes, including alanine aminotransferase (ALT) (Wagner et al., 1986), 5-lipooxygenase (which is a critical enzyme in the production of leukotriene A4 from arachidonic acid) (Wagner and Fessler, 1986; Jahan et al., 2014), trypsin (Syed et al., 2003), Na^+^, K^+^-ATPase (Pocas et al., 2006), and Glucose-6-phosphate dehydrogenase (Luo et al., 2021). WL can also displace [^3^H] flunitrazepam from its binding sites (benzodiazepine sites) on GABA_A_ receptor complex and thus inhibit its binding to the benzodiazepine sites (Pocas et al., 2006). The activities of WL on enzymes and receptors were summarized in Table 4.

**TABLE 4:** Inhibitory activities of Wedelolactone on enzymes and receptors.

Enzymes/Receptors	Concentration	Potential uses	References
IκB kinase	IC_50_ = 10–20 μM	Anti-inflammatory	Kobori et al. (2004)
Caspase-11	IC_50_ = 35 μM	Anti-inflammatory	Kobori et al. (2004)
AChE (acetylcholinesterase)	IC_50_ = 61.23 μg/mL	AD	Phan et al. (2023)
Rat kidney Na^+^, K^+^-ATPase	IC_50_ = 0.7 μM	Heart failure	Pocas et al. (2006)
Benzodiazepine receptor	IC_50_ = 2 μM	Anticonvulsant, anxiolytic	Pocas et al. (2006)
5-lipooxygenase	IC_50_ = 2.5 μM	Anti-inflammatory	Wagner and Fessler (1986)
Glucose-6-phosphate dehydrogenase	IC_50_ = 5.18 μM	Anticancer	Luo et al. (2021)
α-glucosidase	IC_50_ = 39.12 μM	Anti-diabetic	Zhang et al. (2025)
Trypsin	IC_50_ = 2.9 μg/mL	Glomerulonephritis, pancreatitis, burns	Syed et al. (2003)
HIV-1 integrase	IC_50_ = 4.0 μM	Antiviral	Tewtrakul et al. (2007)
NS5B RdRp	IC_50_ = 7–36 μM	Antiviral	(Kaushik-Basu et al., 2008; Tseng et al., 2011; Manvar et al., 2012)
Organic Anion Transporters 1 (OAT1)	IC_50_ = 6.59 μM	Protection against aristolochic acid-induced nephropathy	Li et al. (2020)
OAT3	IC_50_ = 7.09 μM	Protection against aristolochic acid-induced nephropathy	Li et al. (2020)
Organic cation transporter (OCT2)	IC_50_ = 19.14 μM	Protection against Cisplatin-induced nephropathy	Wang et al. (2021)
Tyrosinase	IC_50_ = 1.2 μM	Skin-whitening	Huang et al. (2019)
Nicotinamide N-methyltransferase (NNMT)	IC_50_ = 0.03 μM	Anti-obesity	Yao et al. (2022)
Topoisomerase IIα	100%: 30 μM	Antitumor	Benes et al. (2011)

## 16 Other activities

For hundreds of years, *E. alba* has been used by TCM for curing premature graying of hairs. The promoting activity of hair growth showed by *E. alba* extract (ethanolic and petroleum ether) may be due to the WL within it, which remains to be identified with more well-designed experiments (Roy et al., 2008).

WL can enhance the killing capacity of natural killer (NK) cells through activating JAK/STAT pathway that would increase granzyme B and perforin expression. In addition, WL can also promote NK cell migration through increasing CCR7 and CXCR4 expression (Sun et al., 2023).

WL can also inhibit tyrosinase, and thus lower the production of melanin, indicating its potential use in skin whitening (Huang et al., 2019). Recent publication identified WL as a novel inducer of megakaryopoiesis in K562 and Meg-01 cells. WL can increase the platelet count in mice exposed to X-ray irradiation, without causing systematic damages (Mo et al., 2024). Later, this group revealed that the platelet-increasing effects of WL in radiation-induced thrombocytopenia in mice were mediated through regulation of mitochondrial oxidative phosphorylation and activation of MAPK pathway (Li et al., 2025).

Via activating Keap1/Nrf2/ARE pathway, WL can lower the oxidative stress, apoptosis and autophagy in porcine embryos, thus enhancing the growth and development (Wang et al., 2022). WL can increase the odontoblast differentiation of dental pulp stem cells via semaphorin 3A/neuropilin-1 pathway-mediated β-catenin activation and NF-κB inhibition (Wang et al., 2018).

Besides the beneficial effects of WL in *P. aeruginosa*-induced bacterial keratitis (Xu et al., 2021), *A. fumigatus*-induced fungal keratitis (Cheng et al., 2019), WL can also alleviate the retinal degeneration in mice induced by N-methyl-N-nitrosourea (NMU), through inhibiting Aim2/Casp11 inflammasome activation (Harkin et al., 2022). In addition, WL can alleviate the apoptosis and oxidative stress in human retinal vascular endothelial cells exposed to high glucose through increasing the expression of miR-190 (Cai X. et al., 2024). Thus, WL may have potential usage in treating multiple eye-related diseases.

## 17 Pharmacokinetics and metabolism

Several groups reported the pharmacokinetic profiles of WL after oral administration. The pharmacokinetic parameters in rats and mice have been determined by methods based mainly on high-performance liquid chromatography (HPLC) or ultra‐high‐performance liquid chromatography (UPLC)-coupled with tandem mass spectrometry (MS/MS) (Table 5) (Cheruvu et al., 2018; Jiang et al., 2018; Chen et al., 2019; Xu et al., 2022). The absorption of WL through gastrointestinal tract in rodents is quick, with T_max_ of 0.42–1.93 h while the AUC and C_max_ varied in these studies. The oral bioavailability was predicted to be 49.6% (Qin et al., 2021).

**TABLE 5:** Pharmacokinetic parameters of WL in rat plasma after oral administration.

Subjects	Dose (mg/kg)	Administration route	C_max_ (ng/mL)	T_max_ (h)	T_1/2_ (h)	AUC_0-t_ (ng h/mL)	AUC 0−∞ (ng h/mL)	MRT (h)	References
SD rats	50	Oral	519.37	1.93	3.02	2922.36	3439.99	7.87	Feng et al. (2019)
Wistar rats	0.1	Oral	74.9	0.633	2.20	260.8	261.9	3.24	Xu et al. (2022)
SD rats	10	Oral	17.61	​	2.81	50.47	​	4.35	Sun et al. (2023)
SD rats	5	Oral	15,220	0.5	4.51	​	83.05	4.76	Chen et al. (2019)
SD rats	0.271	Oral	25.62	0.53	2.74	66.27	91.52	​	Du et al. (2018)
SD rats	2.168	Oral	107.37	0.45	2.52	200.91	277.73	​	Du et al. (2018)
SD rats	4.336	Oral	200.72	0.42	2.54	243.52	240.12	​	Du et al. (2018)
SD rats	0.061	Oral	6.39	1.5	2.95	13.77	16.56	3.12	Jiang et al. (2018)
BALB/c mice	0.192	Oral	4.31	1	5.81	27.45	30.08	10.54	Cheruvu et al. (2018)

After oral administration of WL-containing *E. prostrata* extract, WL can also be detected in liver, kidney, spleen, lung and heat (in decreasing order) and the liver is the primary excretion organ (Du et al., 2018), indicating its wide distribution. Another group showed that WL can be detected in brain after oral administration, indicating that it can cross the blood brain barrier (Wei et al., 2023). The tissue distribution is rapid (CL = 0.06 L/h and V = 0.039 L/kg) (Chen et al., 2019). Urine and faces are excretion routes of WL, as WL can be detected in these samples after oral administration (Wei et al., 2023). However, the pharmacokinetic parameters after intravenous administration and the absolute oral bioavailability remains to be determined.

The metabolites of WL in rats have also been explored and the major metabolic pathways were reported to be glucuronidation and methylation (Li et al., 2016; Li et al., 2019). Multiple uridine diphosphate-glucuronosyltransferase were involved in the glucuronidation, which, along with methylation, contributes to the low oral bioavailability in rats (Li et al., 2016).

## 18 Drug delivery

The intestinal absorption of WL is poor and the solubility of WL in water is low (about 0.5), limiting its efficacy (Zhang et al., 2016; Feng et al., 2019; Upadhyay et al., 2012; Sun et al., 2023). To overcome these difficulties, several drug delivery methods were employed. In 2012, Upadhyay et al. complexed WL with phosphatidyl choline and converted them into phyto-vesicles. The bioavailability was increased probably due to the amphiphilic property of the complex that may enhance the solubility and absorption of WL. And the hepatoprotective effects of WL was enhanced by this method (Upadhyay et al., 2012). Zhang *et al* developed a photoresponsive nanodrug delivery system (ICG-liposomal wedelolactone), which enhanced the water solubility and bioavailability of WL (Zhang et al., 2016). This system also provides the on-commend release of WL from carrier under near-infrared light. This method has better efficacy as it achieves synergistic interactions between chemotherapy and photothermotherapy in a cancer xenograft model *in vivo*. This method has WL release of 52.07% at 8 h without light irradiation and 96.74% at 8 h under near-infrared light (Zhang et al., 2016). Later, this research group further designed and synthesized a new delivery system composed of WL-liposome which was encapsulated by gold nanoshell (Au nanoparticles as the surface) (Zhang et al., 2019a). This system improved the traditional disadvantages of liposomes, such as instability (fragility in blood circulation) and stochastic and uncontrolled release. This was also a chemo-photothermal synergistic and on-command release system. This system has not been validated in other diseases besides tumor (Zhang et al., 2019a). In the same year, Feng et al. designed spherically shaped WL-loaded micelles with Solutol^®^ HS15 and lecithin, which demonstrated continuous stability (more than 14 days) and sustained release (Feng et al., 2019). The water solubility was increased to 1.9 mg/mL and the bioavailability was increased by 2.78 fold (Feng et al., 2019). Zhang et al. further used the graphene oxide to develop another trimodal (chemotherapeutic/photothermal/photodynamic) synergistic antitumor drug delivery system. In this system, WL and ICG were co-loaded on the surfaces of graphene oxide through π–π stacking interaction. They can effectively transform optical energy into heat (which facilitates the release of WL) and produce ROS to damage and kill tumor cells. Graphene oxide is nontoxic at certain concentration ranges and biodegradable, making this system low biotoxicity. In addition, by combining with graphene oxide, the photostability of ICG was enhanced. Generally, this system has higher photothermal property and stability (Zhang et al., 2019b). Due to the biodegradable and biocompatible properties, poly (lactide-co-glycolic acid) (PLGA) nanoparticles were employed by Das et al. to encapsulate WL (Das et al., 2019). This formulation of WL could enhance the stability and the retention of WL and thus increasing the biological activity against cancer stem cells that are resistant to conventional chemotherapies. Low toxicity and sustained release were also the advantages of this formulation, which also has improved antibacterial activities (Vinayagam et al., 2021; Das et al., 2019). In another study, WL (as a capping agent) was stabilized on the surfaces of gold nanoparticles in a simple, nontoxic and eco-friendly way and these WL-gold nanoparticles had higher antioxidant activity and improved the insulin secretion function of β cells (Ramachandran et al., 2019). Zhao et al. modified the surfaces of WL-loaded nanoliposomes with pulmonary surfactant protein A monoclonal antibody (SP-A mAb) to enhance the targeting delivery of WL to alveolar epithelial cells (Zhao et al., 2023). These drug delivery strategies remain to be validated in other diseases models.

## 19 Perspective and conclusion

Since its discovery in 1986, WL has demonstrated various kinds of pharmacological activities (Wagner et al., 1986; Wagner and Fessler, 1986). The past 20 years have seen an increase in pharmacological researches on WL. In this review, we summarize the recent pharmacological progresses on WL, which includes antioxidative, antimicrobial, anti-inflammatory, anticancer activities and protective effects on multiple kinds of organs (liver, kidney, lung and bone). The antimicrobial studies on WL lacked enough animal results while many anticancer researches on WL were limited to *in vitro* tests. This coumestan has already been synthesized totally since 2003 (Li et al., 2003; Chang et al., 2008) and a series of derivatives/analogs have also been synthesized (Pocas et al., 2006; da Silva et al., 2001), which made the structure-activity analysis readily. This may further instruct the chemical structural optimization in developing drugs in the future. Although the structural requirement for WL to suppress IKK and caspase-11 activity has been demonstrated to be very stringent (Kobori et al., 2004), this stringency may be less in other pharmacological activities, such as antitumor and antimicrobial activities. More well-designed studies are needed to promote the translation of WL from bench to bedside in the future.

In sum, this article reviews the recent progress of WL in natural sources, pharmacological effects, pharmacokinetics and drug delivery strategies. Some existing problems and future research directions are pointed out, which may benefit the development and translation of WL.
